# Ultrastructural modeling of small angle scattering from photosynthetic membranes

**DOI:** 10.1038/s41598-019-55423-0

**Published:** 2019-12-18

**Authors:** Dainius Jakubauskas, Łucja Kowalewska, Anna V. Sokolova, Christopher J. Garvey, Kell Mortensen, Poul Erik Jensen, Jacob J. K. Kirkensgaard

**Affiliations:** 1 Niels Bohr Institute, University of Copenhagen, DK-2100 Copenhagen, Denmark; 2 Copenhagen Plant Science Centre, Department of Plant and Environmental Sciences, University of Copenhagen, DK-1871 Copenhagen, Denmark; 3 Department of Plant Anatomy and Cytology, Institute of Experimental Plant Biology and Biotechnology, Faculty of Biology, University of Warsaw, Warsaw, Poland; 4 Australian Centre for Neutron Scattering, ANSTO, NSW 2234 Sydney, Australia; 5 Department of Food Science, University of Copenhagen, DK-1871 Copenhagen, Denmark; 6 Biofilm—Research Center for Biointerfaces and Biomedical Science Department, Faculty of Health and Society, Malmö University, Malmö, Sweden; 7 Lund Institute for Advanced Neutron and X-ray Scattering, Lund, Sweden

**Keywords:** Plant sciences, Photosynthesis, Organelles, Chloroplasts, Biological physics, Biomaterials - cells, Soft materials, Self-assembly, Techniques and instrumentation

## Abstract

The last decade has seen a range of studies using non-invasive neutron and X-ray techniques to probe the ultrastructure of a variety of photosynthetic membrane systems. A common denominator in this work is the lack of an explicitly formulated underlying structural model, ultimately leading to ambiguity in the data interpretation. Here we formulate and implement a full mathematical model of the scattering from a stacked double bilayer membrane system taking instrumental resolution and polydispersity into account. We validate our model by direct simulation of scattering patterns from 3D structural models. Most importantly, we demonstrate that the full scattering curves from three structurally typical cyanobacterial thylakoid membrane systems measured *in vivo* can all be described within this framework. The model provides realistic estimates of key structural parameters in the thylakoid membrane, in particular the overall stacking distance and how this is divided between membranes, lumen and cytoplasmic liquid. Finally, from fitted scattering length densities it becomes clear that the protein content in the inner lumen has to be lower than in the outer cytoplasmic liquid and we extract the first quantitative measure of the luminal protein content in a living cyanobacteria.

## Introduction

Photosynthetic electron transfer takes place in the thylakoids - a highly specialised membrane system which in cyanobacteria accommodates both the photosynthetic and cellular respiration protein machinery. In cyanobacteria, several sheet-like thylakoid membranes are arranged as stacks in the cell periphery, confined by the surrounding plasma membrane. The cyanobacterial light-harvesting antennae - phycobilisomes - are densely packed between the thylakoids.

**Figure 1:**
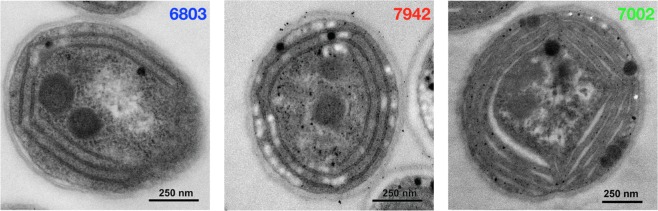
Transmission electron micrographs representative of the typical thylakoid arrangements in the cyanobacterial species investigated. The color code of the three species is maintained throughout the paper.

Cyanobacterial thylakoid arrangements are both species- and strain-dependent^1^ and in Fig. 1 we present thin-section electron micrographs of three biotechnologically important cyanobacterial wild-type strains with varying thylakoid arrangements. In the round *Synechocystis* sp. PCC 6803 cell (6803), 3–6 layers of parallel flattened thylakoids localise in close proximity of the cell membrane and occasionally converge to the thylakoid centers^2,3^. The elongated fresh-water *Synechococcus elongatus* PCC 7942 cell (7942) contains 3–6 layer concentrically arranged thylakoids, which run through the entire cell length without convergence sites^4,5^. The eurhaline *Synechococcus* sp. PCC 7002 cell (7002) has three to four thylakoid stacks, each composed of 3–6 flattened thylakoid sheets, which converge at the edges^6^.

While light microscopy is of insufficient resolution to visualise thylakoids in nanometer resolution^7,8^, 2D transmission electron micrographs as in Fig. 1 can reveal many details of thylakoid membrane organization and the 3D nature of thylakoids can be reconstructed by electron tomography^3,5,6^. However, thylakoid membrane shrinkage occurring from sample fixation and inherently static images limits the applicability of microscopic methods in dynamic studies. By contrast, X-ray and neutron scattering methods are devoid of sample fixation and gives structural information which is a statistical and spatial average of the total irradiated volume of the sample. Thus, scattering and microscopy are complementary methods and can, if properly utilised and combined, provide strong and reliable structural information. For this reason there has been several studies using particularly small-angle neutron scattering (SANS) to gain information on photosynthetic membranes, including cyanobacterial thylakoid membranes^9–14^.

Common to all the SANS studies performed on photosynthetic organisms over the last decade is that the data interpretation and analysis is either based on simple peak position readings or on using some *ad hoc* model expression to extract these peak positions. None of the studies makes use of an underlying structural model to fit the full scattering curve, based on the clear fact that the scattering originates from a stacked membrane system. Neither do they include instrument resolution effects which we will show below to be absolutely necessary for correct data interpretation. Instead, the interpretation of the scattering curves is based on educated guesses regarding peak origin(s) or spuriously treats all peaks as 1^*st*^ order Bragg peaks^10,11,15^.

In this work we formulate a comprehensive structural model, where we account for the double bilayer nature of the photosynthetic membrane stacks, instrumental resolution effects and inherent polydispersity of the system. We validate the structural model by direct scattering pattern simulations using full 3D models and explore model predictions to aid the scattering pattern interpretation.

To extract biophysical parameters of thylakoid membranes, lumenal protein content and to define relevant molecular constraints we perform detailed calculations of scattering length densities from existing knowledge of cyanobacterial cell composition. We demonstrate that the entire neutron scattering curve measured *in vivo* on three different, but structurally typical cyanobacterial thylakoid membrane systems, are fully described within our model framework. Finally, we estimate cyanobacterial lumenal protein content solely from the scattering model.

## Results

### Theory and mathematical modeling

#### Model details

**Figure 2:**
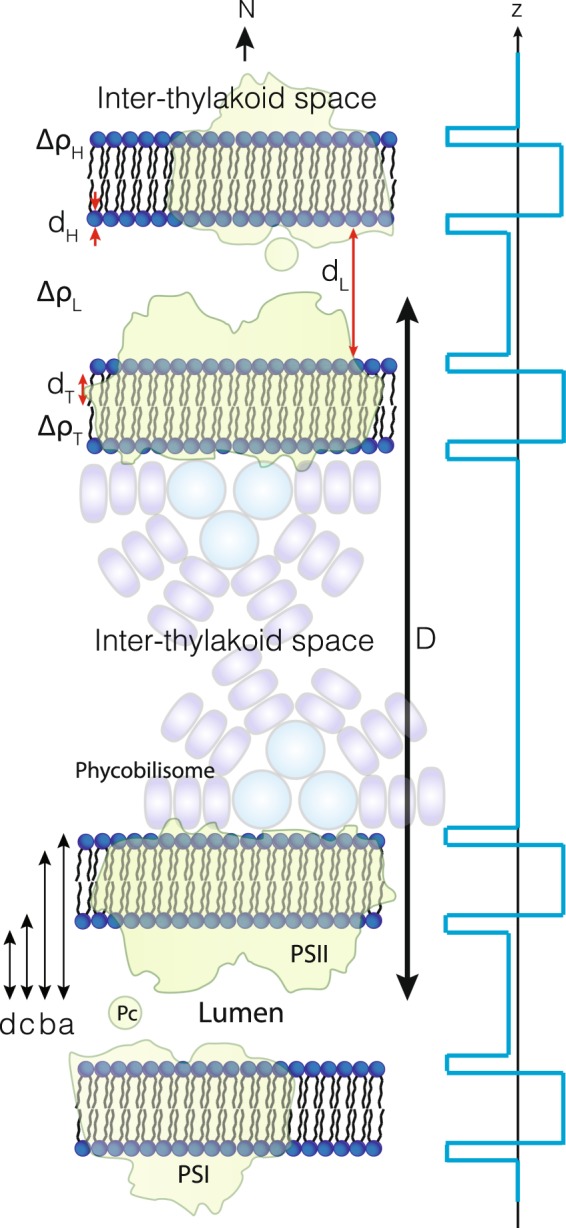
Schematic illustration of the cyanobacterial thylakoid membrane stack and the scattering length density distribution used in the modelling of the scattering data.

We follow the classic work of Nallet *et al*.^16^ on lyotropic liquid crystalline lamellar phases to derive a model expression for the stack. In Fig. 2 we illustrate the basic scattering density profile model of the cyanobacterial thylakoid membrane stack. The difference in our approach is that for the thylakoid membrane stack, the basic building block is not a single lamellar membrane sheet, but a double bilayer thylakoid. Denoting the lamellar repeat distance *D*, the overall model expression for the measured powder averaged intensity takes the form^16^

1 $$I(q)=2\pi \frac{P(q)\cdot S(q)}{D\cdot {q}^{2}}$$

where *P*(*q*) is the unit cell form factor of the thylakoids, ie. the double bilayer, and *S*(*q*) is the structure factor describing the stacking of these unit cells. Assuming Gaussian fluctuations of each layer around an equilibrium position, the lamellar structure factor from Nallet *et al*.^16^ is given by

2 $$S(q)=1+2\mathop{\sum }\limits_{n\mathrm{=1}}^{N-1}\,(1-\frac{n}{N})\cos \,(nq\cdot D)\exp \,(\,-\,{q}^{2}{D}^{2}\alpha (n))$$

with

3 $$\alpha (n)=\frac{{\eta }_{cp}}{4{\pi }^{2}}(\mathrm{ln}\,(\pi n)+{\gamma }_{E})$$

where *N* is the number of lamellae in a stack and *γ*_*E*_ is Euler’s constant. The Caillé parameter *η*_*cp*_ measures the rigidity of the membranes, with a low value indicating a stiff membrane with a high bending modulus, and high values indicating more flexible layers. Setting *η*_*cp*_ = 0 equals perfectly ordered rigid flat sheets which we use below for model validation purposes. The form factor of the double bilayer is built from a series of box functions as illustrated in Fig. 2. With reference to Fig. 2 we now define distances *a*, *b*, *c*, *d* to derive the total double bilayer form factor (see detailed steps in *Materials and Methods F*)

4 $$P(q)=\frac{4}{{q}^{2}}{[\Delta {\rho }_{H}(\sin (qa)-\sin (qb)+\sin (qc)-\sin (qd))+\Delta {\rho }_{T}(\sin (qb)-\sin (qc))+\Delta {\rho }_{L}\sin (qd)]}^{2}$$

with Δ*ρ*_*H*_, Δ*ρ*_*T*_ and Δ*ρ*_*L*_ being the contrasts of the lipid headgroups, lipid tailgroups, and lumen region respectively. Thus, we allow for the lumen to have a scattering length density different from the surrounding cytoplasm liquid. The presented model can be used for both neutron and X-ray data, if contrasts, backgrounds and instrument resolutions are properly accounted for.

#### Model validation, predictions and final formulation

For model validation purposes we employ the brute-force simulation setup previously used to investigate scattering from photosynthetic membrane systems^17^ (see details in *Materials and Methods G*). We have simulated up to 6 double bilayer unit cells (Supplementary Fig. 1). The simulations and the theoretical expressions are in excellent agreement and thus overall validate the derived form factor and the chosen model for the structure factor. Note that there are no fluctuations, polydispersity or instrument resolution invoked in the simulations, all of which influence the experimental scattering patterns, and therefore need to be included in the final analysis of the experimental data.

**Figure 3:**
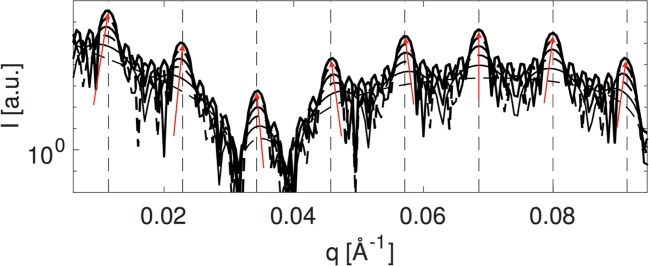
Direct comparison of theoretical scattering patterns corresponding to Supplementary Fig. 1. As the number of unit cells increases from 1 to 6 (plotted with increasing intensity) the manifestation of the Bragg peaks becomes increasingly pronounced. The theoretical position of the Bragg peaks are indicated by dashed lines. The red arrows indicate the curvature of the form factor governing the shift direction of the peaks for few unit cells.

In Fig. 3 we use the theoretical model to compare how the Bragg peaks are manifested in the scattering pattern for the relatively small number of stack layers which are relevant for the cyanobacterial thylakoids (ca. *N* = 3–6). First of all the first order peak requires at least 4 unit cells before the peak maximum appears at the theoretical repeat distance (indicated by the vertical dashed lines in Fig. 3 and Supplementary Fig. 1). This means that the peak positions are modulated by the form factor: in Fig. 3 the direction of this modulation is indicated with red arrows and is clearly a direct reflection of the form factor curvature at each peak position. The most important result of this is that different peaks can move slightly in opposite directions despite clearly originating from the same stacked structure. In relation to the interpretation of the peak positions this can lead to the wrong conclusion that these peaks are uncorrelated stemming from different substructures. Further, for few stack layers the magnitude of the repeat distance can be over- or underestimated depending on which peak is used to describe the system.

**Figure 4:**
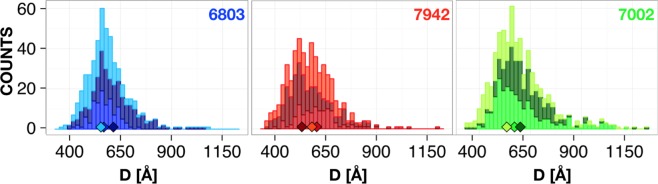
Histograms of repeat distances from TEM measurements of all investigated species (three biological replicas). Diamonds indicate the median of each individual sample.

Another issue which needs to be considered are the effects of instrument resolution and large length scale fluctuations, or polydispersity, on the experimental patterns. It it easy to physically justify the inclusion of polydispersity for the repeat and lumen distances as fluctuations in these parameters are immediately obvious upon inspection of TEM data and is quantified by the statistics shown in Fig. 4. In *Materials and Methods G* we investigate these effects and find that the resolution smearing is absolutely crucial to fully model the data as it has a pronounced effect on the resulting patterns (Supplementary Fig. 2). Further, we find that polydispersity in the repeat distance is equivalent to variations of the Caillé parameter while polydispersity in the lumen distance controls the depth of the pronounced form factor minimum around *q* = 0.01–0.03 Å^−1^ (Supplementary Fig. 3). Thus, in our final model we include lumen polydispersity explicitly while repeat distance fluctuations are included via the Caillé parameter. A final term to be included in the model is a background contribution *I*_*b*_. Although a standard background subtraction of buffer scattering has been performed this does not include the contributions arising from any other biological material present in the samples. There are two main contributions to this, a general flat incoherent background and scattering from the cell wall. As was recently demonstrated the scattering from the latter is basically a *q*^−2^ power law^18^, thus we implement the background contribution as a simple sum of two such terms and add that to Eq. 1: *I*_*b*_ = *B* + *Cq*^−*n*^, where *B*, *C* are constants and where we require the power law exponent *n* to be close to 2. The model is implemented in the WillItFit framework^19^ allowing for instrument resolution effects to be included in the model fits.

#### Molecular constraints

Thylakoid membrane composition differs from other cellular membranes by three aspects: its protein content is significantly higher than other membranes, thylakoid fatty acids are largely non-saturated and thylakoid lipid headgroups are predominantly non-charged galactosides^20–26^. Thus, the scattering length densities calculated for myelin^27^ or artificial lipid membranes^28^ are unsuitable for modelling cyanobacterial or higher plant chloroplast scattering. We calculated average scattering length density values of cyanobacterial thylakoid membrane components to use as molecular constraints or as best estimates for fitting parameters in the modelling. X-ray and neutron scattering length densities were calculated for four different entities: the cyanobacterial inter-thylakoid space and three integral thylakoid membrane components: membrane proteins, lipid headgroups and lipid tailgroups. To calculate the final thylakoid scattering length, these membrane components were averaged using a protein/lipid volume ratio of 0.7/0.3^29^. Chlorophylls and cofactors were not considered in the thylakoid scattering length density calculations because of their low volume fractions. The procedure for these calculations are outlined in *Materials and Methods H* and the results are listed in Supplementary Tables 2 and 3. For convenience during fitting the contrast of tailgroups was constrained to −1 with other values varied relative to that and then subsequently calculated back to absolute units using Supplementary Tables 2 and 3. Also, for the SANS data it became clear that including headgroup scattering as a separate parameter did not improve fittings, therefore the entire membrane thickness was fitted as a single box. Several structural constraints were imposed during the fitting: total membrane thickness (a sum of headgroups + tailgroups) was constrained to 20–60 Å^30–32^, lumen thickness was constrained to 45–300 Å^3,33–35^, repeat distances were constrained to 450–950 Å^1,14^ and the average number of thylakoids was constrained to 2–6^3^.

### Transmission electron microscopy

To assess biological variation we measured thylakoid repeat distances from three biological cyanobacterial replicas of each strain (a total of 1677 measurements, see *Appendix 1*), grown under the same temperature and illumination conditions and fixated during cell exponential phase. Repeat distance (D) distribution histograms (Fig. 4) exhibit some variation within the three replicas. The medians for replicas (diamonds in Fig. 4) are within 530–630 Å range. From a statistical analysis of normalized data, the average D is not significantly different between investigated cyanobacterial species. Therefore, we infer that the average thylakoid repeat distance is the same between 6803, 7942 and 7002 strains and is equal to the average repeat distance of the nine replicas of Supplementary Table 1 yielding ~590 Å.

### Small angle scattering

**Figure 5:**
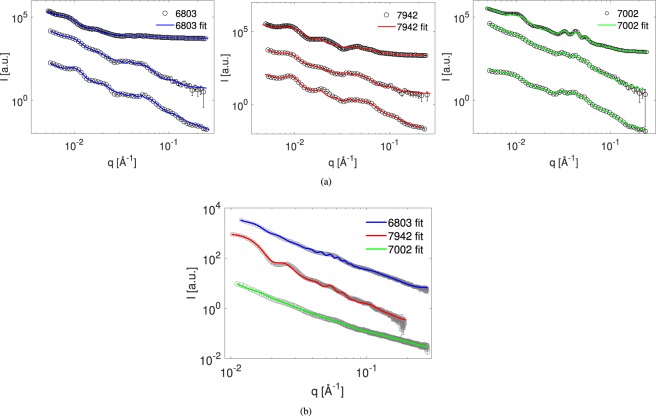
Scattering data and model fits. (**a**) SANS data measured in 100% D_2_O-based media. Each strain is measured in triplicate with replicas 1–3 arranged from top to bottom arbitrarily scaled for clarity. (**b**) SAXS data of these three strains, single replicas.

As with TEM we measured triplicates of SANS data for each of the three cyanobacterial strains. The data from measurements in 100% D_2_O-based medium along with the best model fits are shown in Fig. 5(a). Clear maxima are observed between *q* = 0.01–0.1 Å^−1^, with a group of three distinct maxima around *q* = 0.03–0.05 Å^−1^ best resolved for the 7002 strain. From the model calculations these peaks are identified as clear higher order peaks and thus yield a highly reliable estimate of the overall dominating repeat distance. Otherwise, scattering peaks are largely smeared supporting both the expectation of sample polydispersity and instrument resolution effects. Contrast variation series (Supplementary Fig. 4) in 42% and 21% D_2_O-based medium supports that scattering peaks occur from thylakoid membranes and fits to the background term results in *n* values from 1.95 to 2.5 as expected. As seen from the plots the model fits capture basically all significant features of the data. The model parameters describing the average dimensions of thylakoid system are summarized in Table 1.

**Table 1:** Average structural thylakoid membrane parameters obtained from SANS model fitting.

Fit parameter	6803	7942	7002
*D*, Å	677.8	693.1	597.6
*N*	4	4.3	3.41
*d*_*L*_, Å	62.13	64.26	84.66
*d*_*T*_, Å	16.89	18.46	20.90
Δ*ρ*_*L*_, a. u.	0.334	0.34	0.395
**Deduced parameter**			
*d*_*IT*_, Å	548.1	555.0	429.3
*d*_*TM*_, Å	34	37	42
*SLD*_*L*_, 10^−6^ Å^−2^	4.29	4.30	4.41

Average thylakoid repeat distance of 600–700 Å, number of layers in a thylakoid stack is 3.5–4.5, average lumen thickness is 60–85 Å and average thylakoid membrane thickness is 33.8–42 Å. Inter-thylakoid space height d _*IT*_ = D-d_*L*_-4 · d_*T*_ = 430–555 Å, is in very close agreement with values obtained from (cryo-)TEM studies^1,3,34–36^. From cryoEM measurements, which exclude fixation-induced sample shrinkage, inter-thylakoid space height of 6803 is 580 ± 130 Å, and of 7942–450 ± 30 Å^36^.

The results of SAXS model fits are shown in Fig. 5(b). As seen from the plots, model fit quality is less impressive although the model capture most significant features of the data. In general, the system contrast is lower for X-rays and thus less features are apparent in the data. Experimental smearing is not implemented for the SAXS modeling but will account for the differences around *q* = 0.05 Å^−2^.

Structural parameters obtained from X-ray fits: average thylakoid repeat distance is equal to 460–816 Å, the number of layers in a thylakoid stack is 2–3, average lumen thickness is 63 Å. Derived average thylakoid membrane thickness is 38.5–48 Å and the inter-thylakoid space height d_*IT*_ is 350–710 Å. These values, although slightly higher, are generally comparable to our SANS measurements and we ascribe the difference to the slight differences in the environmental conditions of the two sets of experiments. However, despite the less than perfect X-ray fits, we can still use the SAXS results to discriminate between possible SANS interpretation scenarios.

**Figure 6:**
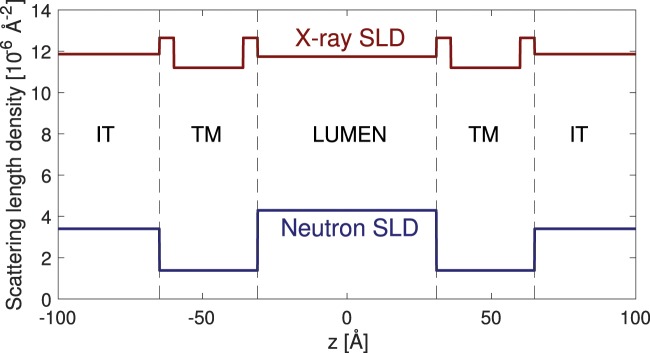
X-ray and neutron SLD profiles of a thylakoid unit cell. Inter-thylakoid space contains 85% protein and 15% of D_2_O.

The scattering length density profile of the thylakoid membrane derived from the fits are shown in Fig. 6 in absolute units. As X-ray and neutron SLDs provide complementary data, the case in which a lumen protein volume fraction calculated from X-ray data is the most similar to lumen protein volume fraction calculated from neutron data is the most likely. Thus, we can estimate the lumen protein content solely from the scattering data provided a few assumptions are met regarding cyanobacterial inter-thylakoid space protein content and their exchange of hydrogen and deuterium. As outlined in detail in *Materials and Methods H* a range of contrast scenarios can be put forward and by solving a set of linear equations the optimal solution can be determined. As observed from Supplementary Table 6, lumenal protein volume fraction is lower than of inter-thylakoid space from both neutron and X-ray calculations. From comparing volume fractions of X-ray and neutron calculations for different scenarios, our best estimate is that lumenal protein content is ~83%, lumenal water volume composition is 90%/10% D_2_O/H_2_O and lumenal protein total hydrogen-deuterium atom exchange is 30%. This result suggests that the lumenal protein content is higher than in the thylakoid membrane, but lower than that of the inter-thylakoid space. To our knowledge, this is the first attempt to quantitatively estimate the protein concentration inside the thylakoid lumen of a living cyanobacteria.

## Discussion

In this paper we have described a straightforward scattering model based on a double-bilayer membrane stack. Our model presents a rationale to explain the entire cyanobacterial scattering pattern as occurring from an ordered lamellar system and we have employed this model to study the ultrastructure of thylakoids inside cyanobacterial cells *in vivo*. Contrary to preceding models, our model is capable of fitting the entire scattering curve from a living biological system. Most importantly, the model fitting yields realistic values of thylakoid membrane thickness, lumen thickness and thylakoid repeat distances. The only other biological scattering model of this kind is of Nickels *et al*.^18^, where the thickness of the *Bacillus subtillis* cell membrane is extracted as a fitting parameter.

Small-angle scattering analyses have previously brought new knowledge in thylakoid membrane research: protein and lipid volume fractions, pigment arrangement in the thylakoid membrane were deduced^28,37^, thylakoid membrane thickness was determined^38^, the hydrophilic nature of both inter-thylakoid space and of thylakoid lumen was also coined from analyzing scattering data^39^. The notion that the first scattering peak occurs from the thylakoid repeat distance has also been suggested, but the origin of the remaining scattering peaks has been unclear^9–13,40–48^.

In this paper we have measured complementary sets of neutron and X-ray scattering on the same cyanobacterial strains. To assess the overall biological variation, three biological replicates of SANS measurement have been performed. As experiments have been carried at different times and cyanobacteria were grown in different white light intensities, the simultaneous fitting and a direct comparison of neutron and X-ray scattering data cannot be performed. However, we have observed that the overall ultrastructural thylakoid parameters obtained from independent SANS and SAXS fittings are comparable and physiologically reasonable. The fittings allowed to construct neutron and X-ray scattering length density profiles of the thylakoid membrane and to hypothesize the composition of its aqueous compartments.

To assess the lumenal composition, numerous scenarios of inter-thylakoid compositions were considered and corresponding lumen compositions were derived. On the basis of comparing fitted neutron and X-ray scattering length densities, we propose an estimate of the lumenal protein volume fraction and conclude that the protein content in the lumen is smaller than in the inter-thylakoid space and is about 83% of the available lumen volume under the applied experimental conditions; available lumenal water volume is 17%, lumen D_2_O/H_2_O ratio is 0.9/0.1. This is, to our knowledge, the first estimate of lumenal protein content in a living cyanobacteria.

To compare, the second-best lumenal composition scenario is that lumenal protein occupies 75% lumen volume, D_2_O/H_2_O ratio is 0.9/0.1. Together, these two scenarios support the argument of Beebo *et al*.^49^, that lumenal water is efficiently exchanged with cytoplasmic (inter-thylakoid space) water. From neutron fits, we also estimate that the exchange of labile H-D of lumenal proteins is 30–50%, what equates to a total protein H-D exchange of 9–15%. The most feasible inter-thylakoid space volume fraction composition resulting from our calculations is 85%/15% phycobilisome/water, of which 13.5% volume is D_2_O and 1.5% is H_2_O. In this case, no exchange of phycobilisome protein labile hydrogens takes place. Similarly, the second-best scenario yields an inter-thylakoid space phycobilisome/water volume fractions of 80%/20% and required labile H-D exchange of phycobilisomes is 0%. In principle, lower H-D exchange of phycobiliproteins compared to lumen proteins is feasible, as phycobiliproteins are large multisubunit complexes, which do not necessarily get fully exchanged, whereas soluble lumenal proteins are generally smaller due to lumen size restrictions and predominantly globular, therefore it is highly feasible that their labile hydrogen can be exchanged to deuterium more easily. Overall, we argue that the complete exchange of inter-thylakoid space water with D_2_O, although not disallowed, is not practically likely, therefore we reject the second-best scenario. In the light of results of Beebo *et al*.^49^, this supports the likelihood of the first composition scenario where both inter-thylakoid space and lumen D_2_O-H_2_O exchanges are high (90%) although incomplete.

An obvious question is how the TEM based size distributions are to be related to the numbers derived from the scattering model which generally show larger values for the dominating repeat distance. The discrepancy lies in the nature of the two methods as the microscopy yields a number averaged distribution (with mean ~590 Å) while the scattering provides volume averaged quantities (with mean ~690 Å). However, transforming a volume averaged log-normal distribution with mean of 690 Å to a number averaged distribution yields a mean of ~590 Å showing that the two methods are in almost perfect agreement (see Supplementary Fig. 5). As mentioned initially, a body of work already exist where scattering methods are used to study photosynthetic membranes^9–14^. We believe that the full modeling approach presented in this paper is superior to previous analysis methods and highlights a number of problems of not having a full structural model. First, our results and modeling shows that the 1^*st*^ order peak is not the most reliable measure of the overall repeat distance as the position of this peak is typically highly affected by the form factor dip. In principle, this means a change in contrast alone could shift the peak without any overall structural changes occurring, particularly for stacks with a low number of layers. The higher order peaks most visible in the 7002 strain are a much more robust measure of this distance but obviously requires one to obtain scattering data of sufficient resolution and quality.

Further, in relation to higher order peaks, it is important to emphasize that assigning for example a 3^*rd*^ order peak as a 1^*st*^ order peak originating from some substructure is erroneous^11^ - our modeling clearly shows that all the peaks can be explained as stemming from one stack with one repeat distance modulated by the form factor of the basic structural unit which is repeated, in this case the double bilayer thylakoids. The same will apply to photosynthetic membranes from other organisms. In fact, the model can naturally be extended to study other organisms - diatoms or systems with functional photosynthetic deficiencies, thylakoid ultrastructure modifications or organisms with larger number of thylakoid layers, such as grana stacks of higher plants. We anticipate that future work will focus on mechanisms of thylakoid ultrastructure adaptation to environmental factors such as thylakoid lumen changes in relation to light intensity and spectral characteristics, different ionic conditions and temperature. The relevance of such new studies based on the modeling approach suggested here will be further enhanced with the superior flux and resolution becoming available at next generation neutron facilities.

## Materials and Methods

### A. Cyanobacterial strains and growth conditions

Strains used in this experiment: *Synechocystis* sp. PCC 6803, *Synechococcus elongatus* PCC 7942 and *Synechococcus* sp. PCC 7002. For growth of cyanobacteria on plates, liquid BG-11 or A + (supplemented with 4 mg/L vitamine B_12_) growth medium was supplemented with 15 g/L bactoagar and autoclaved; plates were kept at 30 °C at 50 *μ*mol photons m ^−2^ s ^−1^. Cyanobacterial liquid cultures for TEM and SAXS experiments were grown in 20 mL flasks at 30 °C at 50 *μ*mol photons m ^−2^ s ^−1^ white light, flasks were shaken 150 rpm. Cyanobacterial liquid cultures for SANS experiment were grown in the same conditions, but light intensity was 5–6 *μ*mol photons m ^−2^ s ^−1^. When a cell culture reached a logarithmic phase OD_730 *nm*_ = 0.8–2, cells were centrifuged (5000 g, 5 min), resuspended in a small amount of fresh 100% D_2_O BG-11 or A+ medium and subjected for scattering experiments.

### B. Small angle neutron scattering

SANS measurements were performed at the Bilby beamline in ANSTO, Sydney, Australia, which was operated in time-of-flight data collection mode^50^. For SANS measurements, cyanobacterial cells were centrifuged and pellet was resuspended in 21, 42, 85 or 100% (v/v) D_2_O-based media, final OD_730 *nm*_ was adjusted to 2.2. Cyanobacteria were measured in 1 mm pathlength demountable cells with quartz windows, ambient illumination was 2–3 *μ*mol photons m ^−2^ s ^−1^ white light.

Neutron beam was defined by 15 mm radius sample aperture, source-sample distance 12.77 m, sample-detector distance 10 m, beamstop radius 40 mm. Data collection time − 1.5 to 3 hours. Neutrons with a range of wavelengths from 4 to 14 Å were used to cover a *q*-range from 0.0015 to 0.38 Å^−1^; *q*-range from 0.0015 to 0.26 Å^−1^ has been taken into account, because wider angles data were not distinguishable from background. Data reduction was performed using Mantid software^51^. The data are shown as plots of the absolute intensity I versus the wave vector *q* = 4 *π* sin(*θ*/2)/*λ*, where *λ* is the wavelength of neutrons and *θ* is the scattering angle. Corrections for background scattering, shape of the incident spectra and the absolute calibration were performed using transmission and scattering measurements of the empty beam, blocked beam, empty cell and corresponding buffers.

### C. Small angle X-ray scattering

OD_730 *nm*_ of exponential cyanobacterial liquid cultures was adjusted to 2, cells were pelleted (5 min, 5000 g) and resuspended in 150 *μ*L fresh growth medium. Small angle X-ray scattering of cyanobacterial cell suspension was measured using 0.15 mm diameter quartz capillaries with GANESHA instrument (SAXSLAB, Denmark) at University of Copenhagen. GANESHA instrument was equipped with 40 W micro-focus Cu source Micromax 002+ of *λ* = 1.54 Å (Rigaku) and 300k Pilatus pixel detector. Sample-detector distances measured in: 690 and 1490 mm, photon fluxes: 62 or 17 · 10^6^ photons/s, covered *q* range: 0.007–0.4 Å^−1^. Silver behenate standard was used for *q* calibration, scattering data reduction was carried out using SAXSgui platform.

### D. Transmission electron microscopy

Cyanobacterial cells were centrifuged and the pellet was fixed in 2.5% (w/v) glutaraldehyde and 50 mM cacodylate in either BG-11 or A+ media for 4 h (room temperature), washed with 50 mM cacodylate buffer (pH 7.4) and placed in a 2% (w/v) OsO_4_ at 4 °C in 50 mM cacodylate buffer (pH 7.4) for about 12 h. The specimens were dehydrated in graded acetone series, embedded in a low viscosity epoxy resin and cut on a Leica EM-UC7 ultramicrotome. Thin sections stained with uranyl acetate were examined with a JEM 1400 electron microscope (Jeol, Japan) in the Laboratory of Electron Microscopy, Nencki Institute of Experimental Biology of PAS, Warsaw, Poland. Repeat distances of cyanobacterial thylakoids have been manually measured in ImageJ software from TEM images of cyanobacterial cells.

### E. Statistical analysis

Repeat distances were measured on TEM micrographs of cyanobacterial cells using the ‘Measure’ function in ImageJ 1.40 g^52^. Statistical comparisons of average repeat distance differences in different cyanobacterial species with respective replicas (total of 1677 measurements) were performed in *R* software. A number of thylakoid stacks was measured for each strain and each replicate (Supplementary Table 1). Data normality was checked by Shapiro-Wilk test and by evaluating residual Q-Q plots. Since tests indicated residual deviations from normality, Box-Cox transformation of data (*λ*_*Box*−*Cox*_ = −2/3) had been performed to normalize data for statistical analysis. D outliers were not omitted from data analysis. To compare average D differences between cyanobacterial species, a linear mixed model on Box-Cox transformed data with repeat distance as a quantitative variable, Species as qualitative variable and Replicas as a random effect has been derived. *post hoc* Tukey contrast test with 0.95 confidence level showed, that average repeat distance values within three species are not significantly different (p > 0.5). D average, median, standard deviation values and mean confidence limits (given in Supplementary Table 1) were calculated in *R* on non-transformed data using lsmeans package. The average D value of all cyanobacterial species calculated in this paper − 590 Å - is the global average of nine individual repeat distance replicas.

### F. Derivation of double bilayer form factor

Denoting the direction normal to the membrane planes *z*, the scattering length density of a single box of thickness 2*δ* is

5 $${\rho }_{0}(z)=\Delta \rho \,{\rm{for}}\,-\,\delta \le z\le \delta $$

and zero elsewhere where Δ*ρ* is the contrast of the material represented by the box, i.e. Δ*ρ* is the scattering length density difference relative to some chosen reference, in this case the surrounding cytoplasm (inter-thylakoid space) liquid. The scattering amplitude is the Fourier transform of the scattering length density function

6 $$A(q)=\int \,{\rho }_{0}(z)\,\exp \,(iqx)dx$$

and the form factor is the absolute square of the amplitude

7 $$P(q)=|A(q{)|}^{2}.$$

To construct the full unit cell we exploit that we can add and subtract boxes on the amplitude level to build up a double bilayer profile. The Fourier transform of a single box of width 2*δ* is

8 $${A}_{box}(q)={\int }_{-\delta }^{\delta }\,\Delta \rho \,\exp \,(iqx)dx=\frac{\Delta \rho }{q}2\,\sin \,(q\delta )$$

giving the box form factor

9 $${P}_{box}(q)=\frac{\Delta {\rho }^{2}}{{q}^{2}}4\,{\sin }^{2}(q\delta )$$

With reference to Fig. 2 in the main text we define the distances *a*, *b*, *c*, *d* as follows

10 $$\begin{array}{rcl}a & = & {d}_{L}\mathrm{/2}+2{d}_{T}+2{d}_{H};\\ b & = & {d}_{L}\mathrm{/2}+2{d}_{T}+{d}_{H};\\ c & = & {d}_{L}+{d}_{H};\\ d & = & {d}_{L}\mathrm{/2;}\end{array}$$

to derive the total double bilayer form factor, Eq. 4 in the main text.

### G. Simulation of scattering patterns and fitting procedure

The simulations consists of calculating the Debye equation

11 $$I(q)=\mathop{\sum }\limits_{i\mathrm{=1}}^{N}\mathop{\sum }\limits_{j\mathrm{=1}}^{N}{b}_{i}{b}_{j}\frac{sin(q{r}_{ij})}{q{r}_{ij}}$$

for a specified *N*-point cloud with scattering lengths *b*_*i*_ and separations *r*_*ij*_ representing the sample as described in^17^. The membranes are represented as large discs with a radius of 1000 Å so any effects of the finite disc size is outside the *q*-range in question. In the simulations 200 bins are distributed along the *q*-axis within a *q*-range of 0.005 Å^−1^ and 0.3 Å^−1^ matching the experimental range. For each *q* value the Debye sum is calculated using a parallel Matlab-based code. Each unit cell is built from 140000 random points. Because of the brute force nature of the simulations, the calculations become prohibitively expensive for large number of points. As mentioned in the main text, the agreement is excellent with the exception of discrepancies at the form factor minima, where the simulations are particularly sensitive to numerical error and the noise inherent in a finite point Monte Carlo based setup (note that the intensity scale is logarithmic).

In Supplementary Fig. 2 we compare model scattering patterns with and without the instrumental resolution smearing from the Bilby instrument. It is clear that for SANS data the resolution smearing is absolutely crucial to fully model the data as it has a pronounced effect on the resulting patterns. Further, we compare the model predictions for three values of the Caillé parameter going from a perfect flat sheet (*η*_*cp*_ = 0) to very stiff layers (*η*_*cp*_ = 0.01) and finally more undulated sheets (*η*_*cp*_ = 0.1). This progression leads to a gradual smearing of peak features from the high *q* end of the spectrum. The conclusion is that the appearance of the very distinct peak series around *q* = 0.05–0.08 Å^−1^ which is clear in the experimental data in Fig. 5 will be a signature of a stiff and well-ordered membrane system and will pose a requirement to keep the Caillé parameter very low. On the other hand, the local membrane thickness is hard to detect so we do not include polydispersity for this. An important feature when comparing the model calculations in Supplementary Fig. 2 and the experimental curves in Fig. 5 in main text is that the pure model displays a very deep form factor minimum around *q* = 0.01–0.03 Å^−1^.

However, from the experimental data it is clear that the peaks in this region still survive although to a varying degree. In Supplementary Fig. 3 model calculations illustrating the effect of polydispersity on the repeat distance and lumen width are shown. The polydispersity is simply implemented as an sum across a Gaussian distribution, thus the repeat distance and lumen width each becomes associated with a standard deviation *σ*_*D*_ and *σ*_*L*_. In the implementation of the double polydispersity one can do the full double sum over the two distributions, but it turns out that one can split the two effects with negligible effect (tested, not shown), i.e. one can calculate the polydisperse form factor first (lumen polydispersity) and then use that in the sum over the structure factor distribution (repeat distance polydispersity) speeding up the fitting routine. As illustrated in Supplementary Fig. 3 lumen polydispersity smears out the deep form factor minimum and smears out structure factor features for ca. *q* > 0.1 Å^−1^. Repeat distance polydispersity on the other hand smears peaks slightly, but mostly for *q*-values higher than the deep form factor minimum which is hardly affected. It maintains the 3 peak features around *q* = 0.3–0.5 Å^−1^ for quite large polydispersity values, but smears out features for higher *q* values. Not surprisingly, the effect of repeat distance fluctuations are basically the same as increasing the Caillé parameter. The combined effect of simultaneous variation of the overall repeat distance and the lumen basically smears all high *q* features leaving only broad 1^*st*^ and 2^*nd*^ order peaks.

The procedure for conducting the fits is not easily transcribed in a single sentence. The most general description would be to first determine the repeat distance which is fairly accurately determined from the higher order peaks around *q* ≈ 0.05 Å^−1^. Also, starting out with representing the bilayer as one large box allows a rough determination of the bilayer width, lumen width and associated contrasts as these parameters to a large extent control the peak ratios of the characteristic three peak pattern in this region. After that parameters are tweaked into place followed by a final global fit. The fit uses a combination of the Levenberg-Marquardt algorithm for quick adjustments of single parameters but more generally the grid search implementation of the algorithm which is implemented in the WillItFit framework^19^. The WillItFit instrument resolution implementation follows Pedersen *et al*.^53^. Also, care has been taken in the WillItFit development to implement trust region estimation, based on the profile likelihood strategy of Pedersen *et al*.^19^. The resulting fit parameters are listed in Table 1 in main text and Supplementary Tables 4 and 5.

### H. Scattering length density calculations

We briefly re-iterate the labelling system which is heavily used in this section. Subscripts are short for the following membrane entities: H - lipid headgroups, T - lipid tailgroups, P - protein, TM - full thylakoid membrane, L - lumen, IT - inter-thylakoid space (cyanobacterial cytoplasm). Further labelling by N or X distinguishes neutron and X-ray scattering length densities - SLD’s.

#### H.1. Thylakoid membrane proteins

To avoid individual protein volume calculations and their multimerization, amino acid sequences of all unique subunits of *T. vulcanus* PSII (PDB ID: 4UB6, 20 unique subunits), *S. elongatus* PSI (PDB ID: 1JB0, 12 unique subunits), *S. cerevisiae* V-ATPase (PDB ID: 3J9T, 11 unique subunits) and *M. laminosus* cytochrome b_6_*f* (PDB ID: 4H13, 8 unique subunits) were subsequently joined into four long polypeptide sequences. Neutron SLDs of these polypeptides were individually calculated using the Biological Scattering Length Density Calculator with standard parameters and amino acid volumes^54^ in 100% D_2_O. As photosynthetic complexes are large membrane-embedded proteins and cyanobacterial cell equilibration time in D_2_O-media is maximally two hours, 0% labile H-D exchange in thylakoid membrane-embedded proteins^55^ was considered. Individual polypeptide SLDs were average-weighted using their protein molar ratios (1 PSII:0.7 PSI:0.7 Cyt *b*_6_*f*:0.5 ATPase^56^) and used in the SLD_*T*_ and SLD_*H*_ calculations. Protein density of 1.33–1.35 g/mL^57^ was used.

#### H.2. Thylakoid membrane lipids and saccharides

Lipids constitute 10–28% of cyanobacterial dry weight^58,59^. Although the major lipid classes of cyanobacteria are similar to plants and algae^60^, the fatty acid compositions of their lipids differ^59^, as cyanobacterial thylakoid membranes do not contain polyunsaturated fatty acids^23^. Cyanobacterial lipid SLD was calculated with the NIST scattering length density calculator separately for individual fatty acids (tailgroups) and sugars (headgroups), using fatty acid composition of *Synechocystis* sp. PCC 6803^23,61^ and physical properties of respective individual fatty acids and sugars. Then, a composition-weighted mixture of respective 16 and 18-C fatty acids was used for SLD_*T*_ calculations. Similarly, a composition-weighted mixture of galactose, sulfogalactose and phosphoglycerol was used in SLD_*H*_ calculations.

#### H.3. Thylakoid membrane final SLD

During the fitting of the SANS data it became clear that the neutron contrast does not allow us to distinguish between membrane headgroup and tailgroup regions, thus for the neutron model we describe the whole thylakoid membrane as a single scattering length density box. To calculate the average SLD_*TMN*_ value of this box, SLD_*TN*_ and SLD_*HN*_ were averaged with SLD_*PN*_ using a ratio 0.7/0.3 as mentioned above. The final SLD_*TMN*_ value is then defined as a sum of 1/2 protein-averaged SLD_*T*_ and 1/2 protein-averaged SLD_*H*_, thus: SLD_*TMN*_ = 0.5 · (1.832 · 10^−6^ Å^−2^ + 1.327 · 10^−6^ Å^−2^) = 1.58 · 10^−6^ Å^−2^. This value was used throughout the analysis of the SANS data (Supplementary Tables 2 and 3). In the case of X-ray scattering, such averaging is not performed. Both SLD_*HX*_ and SLD_*TX*_ are used individually in fitting.

#### H.4. Inter-thylakoid space SLD

For simplicity, we assume that inter-thylakoid space is only composed of phycobilisomes and heavy water. Using *Porphyridium cruentum* phycobilisome dimensions and phycobilisome packing density in low light^62^ and the average inter-thylakoid space width of 590 Å, obtained from Supplementary Table 1, we obtain that the phycobilisome volume fraction in the inter-thylakoid space varies from 68–100%. Therefore, the average volume fraction of 60–85% phycobilisomes and 40–15% D_2_O was used in SLD_*IT*_ calculations in Supplementary Table 6. To calculate SLD_*phycobilisome*_, 25 unique protein chains of *Griffithsia pacifica* phycobilisome (PDB ID: PY6P)^63^ were combined into a single polypeptide and calculated as described in the H.1. section. Since phycobilisomes have a higher water accessibility than thylakoid membrane proteins, we assume that their labile H-D exchange is similar to globular proteins and that 0–90% of exchangeable protein hydrogens are exchanged to deuterium, which effectively amounts to 0–27% of all protein hydrogens exchanged to deuterium^64^.

#### H.5. Lumen SLD

The exact lumen protein content is unknown^65^. Therefore lumen composition is assumed to be a mixture of plastocyanin and D_2_O with their respective volume fractions. That is, in SLD_*L*_ calculations, all lumenal proteins - as they are small and mainly globular - are together accounted as plastocyanin (we refer to it as ‘relative plastocyanin’) dissolved in D_2_O. It is estimated that all lumenal water is exchanged by the cytoplasmic water 100 times per second^49^, therefore we assume that after three subsequent cyanobacterial resuspension cycles in 100% D_2_O, cyanobacterial lumen contains predominantly heavy water. We do not account for any spatial or temporal variation in the SLD_*L*_ due to illumination-induced ion transport and resulting lumen volume changes^34^, as the cyanobacteria were not illuminated during the scattering measurements.

#### H.6. From SLD to contrast

Object scattering in a solvent only arises if the scattering density difference between the object and the solvent is non-zero. In this paper, we define the thylakoid membrane as ‘the object’ and the inter-thylakoid space as ‘solvent’, i.e. we calculate thylakoid and lumen contrasts relatively to the inter-thylakoid space. For convenience and to minimize the number of fitting parameters, we arbitrarily define the SLD_*T*_ as −1. That is, we assume that relatively to the inter-thylakoid space, the tailgroup scattering is lower and the contrast between inter-thylakoid space and tailgroups is set to 1 arbitrary unit. Lumen contrast is scaled accordingly by constants *C*_1_ and *C*_2_, which are derived from fits and all numbers are subsequently mapped back to absolute values. The absolute value of SLD_*TM*_ is fixed to 1.58 · 10^−6^ Å^−2^ and since we define contrast relative to the inter-thylakoid space, the contrast for this is obviously 0. For the inter-thylakoid space (SLD_*IT*_) the range of absolute values is 3.61–4.43 · 10^−6^ Å^−2^ (neutron) and 11.16–11.9 · 10^−6^ Å^−2^ (X-ray) (Table 1, Supplementary Table 2, Fig. 3). To obtain scattering contrasts of thylakoid and lumen, we subtract SLD_*IT*_ from SLD_*TM*_ and SLD_*L*_. This assumption has an underlying physical explanation. Firstly, absolute SLD_*IT*_ values cannot be calculated precisely due to unknown protein composition and concentration. If so, derivation of absolute SLD_*L*_ value is prone to large errors. Secondly, scattering length densities relative to inter-thylakoid space (in arbitrary units) are obtained from scattering curve fittings (Supplementary Tables 2, 3 and 5) and can be converted into absolute scale under the assumption that thylakoid membrane and inter-thylakoid space composition are known (see below). Thirdly, the relative SLD_*L*_ comparison to SLD_*IT*_ allows calculating the SLD_*L*_ value on absolute scale. Combining information from X-ray and neutron fits and varying inter-thylakoid space composition, we estimate volume fractions of lumenal D_2_O and total lumenal protein (expressed as the ‘relative plastocyanin’, see Section H.5.).

#### H.7. Lumen protein volume fraction

Absolute values of *SLD*_*LX*_ and *SLD*_*LN*_ have been calculated from the relative average values obtained from fittings (X-rays: −0.175, neutrons: 0.34) using different inter-thylakoid space volume compositions - i.e. varying phycobilisome/water volume fractions, different labile H-D exchange percentage and D_2_O/H2O fraction inside inter-thylakoid space. The scattering length density profile of the thylakoid membrane with absolute values is depicted in Fig. 6 in the main paper (values are given in Supplementary Table 3). Total lumenal protein, expressed as ‘relative plastocyanin’ was calculated from absolute values of *SLD*_*LX*_ and *SLD*_*LN*_, solving the system of coupled equations in Eq. 12.

12 $$\{\begin{array}{l}\begin{array}{rcl}SL{D}_{I{T}_{N}} & = & \frac{{\phi }_{{{\rm{D}}}_{2}{\rm{O}}}}{{\phi }_{{{\rm{H}}}_{2}{\rm{O}}}}\cdot SL{D}_{wate{r}_{N}}+{\phi }_{phycobilisom{e}_{N}}\cdot SL{D}_{phycobilisom{e}_{N}w \% labileH-Dexchange}\\ SL{D}_{I{T}_{X}} & = & {\phi }_{water}\cdot SL{D}_{wate{r}_{X}}+{\phi }_{phycobilisom{e}_{X}}\cdot SL{D}_{phycobilisom{e}_{X}}\\ SL{D}_{{L}_{N}} & = & \frac{{\phi ^{\prime} }_{{{\rm{D}}}_{2}{\rm{O}}}}{{\phi ^{\prime} }_{{{\rm{H}}}_{2}{\rm{O}}}}\cdot SL{D}_{wate{r}_{N}}+{\phi }_{plastocyani{n}_{N}}\cdot SL{D}_{plastocyani{n}_{N}w^{\prime}  \% labileH-Dexchange}\\ SL{D}_{{L}_{X}} & = & {\phi ^{\prime} }_{water}\cdot SL{D}_{wate{r}_{X}}+{\phi }_{plastocyani{n}_{X}}\cdot SL{D}_{plastocyani{n}_{X}}\\ SL{D}_{{L}_{N}} & = & {C}_{1}\cdot SL{D}_{I{T}_{N}}\\ SL{D}_{{L}_{X}} & = & {C}_{2}\cdot SL{D}_{I{T}_{X}}\end{array}\\ \begin{array}{rcl}{\phi }_{phycobilisome}+{\phi }_{water} & = & 1\\ {\phi }_{plastocyanin}+{\phi ^{\prime} }_{water} & = & 1\end{array}\end{array}$$

Proportionality constants *C*_1_ and *C*_2_ were derived from fits, SLD_*IT*_ and SLD_*L*_ were calculated for several scenarios: with different protein/water volume fractions, for the case of neutrons also varying D_2_O/H_2_O volume fractions of total water composition, and with different protein labile H-D exchange percentages (*w*). Lumen protein volume fractions, derived for a number of IT composition scenarios are given in Supplementary Table 6.

As X-ray and neutron SLDs provide complementary data, the case in which a lumen protein volume fraction calculated from X-ray data is the most similar to lumen protein volume fraction calculated from neutron data is the most likely. Such conditions are denoted in orange in Supplementary Table 6, the best fit is denoted in green, second-best in brown. It is also assumed that ${\phi ^{\prime} }_{{D}_{2}O}$ in the lumen is either equal or lower to the inter-thylakoid space ${\phi }_{{{\rm{D}}}_{2}0}$.

Again, scattering contrast Δ*ρ*_*T*_ is constrained to −1 and SLD_*TX*_ absolute value is fixed to 11.2 · 10^−6^ Å^−2^. Contrary to neutrons, headgroup scattering was included as a separate parameter in SAXS modelling (therefore no SLD_*TMX*_ is calculated). Accordingly, thylakoid membrane thickness in SAXS model was a double sum of tailgroup and headgroup thicknesses. The model parameters describing the average dimensions of the thylakoid system are summarized in Table 1. Average SLD_*HX*_ value obtained from fits is slightly lower than purely theoretically calculated (11.9 vs. 13.4 · 10^−6^ Å^−2^, Supplementary Table 2), but this difference is acceptable. Compared to earlier literature value of average SLD_*TMX*_ = 400 electrons/nm^3^ = 7.27 · 10^−6^ Å^−2^ (50% protein, 30% lipid), SLD_*HX*_ = 450 electrons/nm^3^ = 8.18 · 10^−6^ Å^−2^, SLD_*TX*_ = 160–280 electrons/nm^3^ = 2.91–5.09 · 10^−6^ Å^−2^ from Hodapp *et al*.^66^, SLD_*X*−*ray*_ values derived in this article are slightly higher - most likely due to a higher protein content in the thylakoid membrane than used in Hodapp *et al*., but largely comparable.

## Supplementary information

Supplementary material
