# Supplementary material for: Ultrastructural modeling of small angle scattering from photosynthetic membranes
Source: Sci Rep. 2019 Dec 18;9:19405. doi: 10.1038/s41598-019-55423-0 (PMC6920412; doi:10.1038/s41598-019-55423-0)
Supplement: Supplementary file 1 — Supplementary material [file 41598_2019_55423_MOESM1_ESM.pdf]

Ultrastructural modeling of small angle scattering from photosynthetic membranes

Dainius Jakubauskas[1,2], Łucja Kowalewska[3], Anna V. Sokolova[4], Christopher J. Garvey[4], Kell Mortensen[1], Poul Erik Jensen[2], and Jacob J. K. Kirkensgaard[1,5*]

[1]Niels Bohr Institute, University of Copenhagen, DK-2100, Copenhagen, Denmark
[2]Copenhagen Plant Science Centre, Department of Plant and Environmental Sciences, University of Copenhagen, DK-1871, Copenhagen, Denmark
[3]Department of Plant Anatomy and Cytology, Institute of Experimental Plant Biology and Biotechnology, Faculty of Biology, University of Warsaw, Warsaw, Poland
[4]Australian Nuclear Science and Technology Organisation, NSW 2234, Sydney, Australia
[5]Department of Food Science, University of Copenhagen, DK-1871, Copenhagen, Denmark
[*]jjkk@food.ku.dk

ABSTRACT

Supplementary Information

Supplementary Table 1. Cyanobacterial repeat distances (D), measured from TEM micrographs. CL are 0.95 confidence limits of average repeat distances of the individual replicate.

Strain	Replica	Number of thylakoid stacks	Average D, Å	Median D, Å	Standard deviation	Lower CL	Upper CL
6803	1	114	574.1	568.1	10.91	552.7	595.5
	2	198	632.5	615.0	8.28	616.3	648.8
	3	189	556.9	554.2	8.48	540.3	573.6
7942	1	114	612.8	605.5	10.91	591.4	634.2
	2	207	565.7	532.6	8.10	549.8	581.6
	3	204	572.7	582.1	8.16	556.6	588.7
7002	1	242	615.8	572.6	7.49	601.1	630.5
	2	205	622	601.1	8.14	606.0	638.0
	3	204	556.7	534.6	8.16	540.7	572.7

Supplementary Table 2. Absolute SLD values of cyanobacterial components. Values for aqueuous compartments (lumen and inter-thylakoid space) are calculated, estimating 60-85 % (v/v) phycobilisome content in the inter-thylakoid space.

	Neutron SLD, 10^{-6} Å^{-2}	X-ray SLD, 10^{-6} Å^{-2}
SLD_H	1.83	13.4 (calculated)<br>11.9 (from fits)
SLD_T	1.33	11.2
SLD_{TM}	1.58	12.3
SLD_L	5.40-4.30	11.16-11.74
SLD_{IT}	4.43-3.61	11.16-11.86
SLD_{D_2O}	6.4	9.47

Supplementary Table 3. Absolute SLD values of inter-thylakoid space and lumen.

Protein/Water vol. fraction, %/%	D_2O/H_2O, %/%	Labile H-D exchange, %	Neutron SLD, 10^{-6} Å^{-2}	X-ray SLD, 10^{-6} Å^{-2}
SLD_{IT}				
60/40	100/0	0	3.69	11.16
60/40	100/0	90	4.432	11.16
70/30	100/0	0	3.23	11.44
80/20	100/0	0	2.78	11.72
85/15	100/0	0	2.55	11.86
85/15	90/10	0	2.45	11.86
85/15	90/10	90	3.40	11.86
85/15	100/0	90	3.612	11.86
SLD_L				
60/40	100/0	0	4.40	11.16
60/40	100/0	90	5.40	11.16
70/30	100/0	0	4.96	11.40
80/20	100/0	0	3.19	11.63
85/15	100/0	0	2.89	11.75
85/15	90/10	0	2.75	11.75
85/15	90/10	90	4.02	11.75
85/15	100/0	90	4.30	11.75

Supplementary Table 4. Individual fitting parameters for SANS profiles of cyanobacteria, three experimental replicates.

	6803			7942			7002		
	1	2	3	1	2	3	1	2	3
$\Delta\rho_T$, a.u.	-1	-1	-1	-1	-1	-1	-1	-1	-1
$\Delta\rho_L$, a.u.	0.47±0.046	0.33±0.044	0.20±0.025	0.5±0.038	0.29±0.04	0.27±0.019	0.31±0.023	0.48±0.055	0.39±0.019
d_T, Å	13.83±0.60	17.84±0.91	19.02±0.43	20.17±0.5	17.14±0.93	18.08±0.34	19.85±0.50	23.26±1.17	19.60±0.44
d_L, Å	53.04±3.18	72.42±4.17	60.96±1.50	47.57±1.83	80.53±5.54	64.42±1.41	87.00±1.96	80±3.73	87±1.71
RD, Å	780.3±33.9	643.2±15.39	610.11±5.09	704.50±11.35	699.1±22.0	675.85±5.17	586.01±4.77	588.24±9.68	618.52±6.37
N	6±1.12	3±0.92	3±0.36	6±3.97	3.0±0.99	4.06±0.55	5.97±3.9	2±0.583	2.282±0.36
η_{cp}	0.029	0.02	0.02	0.0266	0.029	0.0292	0.02	0.018	0.0309
Background (B)	0.55±0.01	0.0044±0.0042	0±0.006	0.22±0.0072	0.005±0.004	0±0.0067	0.069	0.0015	0
Scale	6.6e-8±6.3e-9	1.4e-8±1.7e-9	1.9e-7±8.5e-9	6e-8±3.1e-9	9.7e-9±1.4e-9	2.4e-7±8.9e-9	5.8e-8±2.9e-9	1.8e-8±1.5e-9	1.6e-7±6.5e-9
σ_L, Å	17.61±4.11	7.30±13.88	8.56±3.20	20.83±2.02	13.28±12.64	13.92±2.03	16.65±2.80	15.23±6.03	19.30±1.98
n power law	2.55±0.018	2.543±0.00837	2.35±0.006	1.93±0.01	2.22±0.016	2.19±0.007	2.09±0.0081	2.534±0.0068	2.25±0.0069
Scale power law (C)	2.1e-5±1.6e-6	2.5e-5±9.8e-7	7.5e-4	4e-4±2.9e-6	4e-5±2.9e-6	9e-4±2.4e-5	4e-4±1.31e-5	5.6e-6±1.68e-6	5.1e-4±1.45e-5
χ^2	5.25	7.51	64.45	80.9	1.95	69.25	36.25	11.31	24.34

Supplementary Table 5. Individual fitting parameters for SAXS profiles of cyanobacteria, one experimental replicate.

	6803	7942	7002
		Fit parameters	
$\Delta\rho_H$, a. u.	1.32±0.044	1.07±0.07	1.20±0.08
$\Delta\rho_T$, a. u.	-1	-1	-1
$\Delta\rho_L$, a. u.	-0.02±0.0144	-0.47±0.0144	-0.035±0.0312
d_H, Å	6.0±0.2	4.65±0.25	6.95±0.45
d_T, Å	15.8±0.22	19.2±0.34	12.3±0.44
d_L, Å	62.5±2.75	63.7±2.71	63.8±12.63
RD, Å	815.8±4.63	462.8±2.61	748.1±30.8
N	2±0.23	3±0.28	2±1.03
σ_L, Å	18.0±2.5	15.0±3.28	19.5±9.53
η_{cp}	0.01	0.036	0.02
Background (B)	0.03±0.002	0.024±0.0043	0.020±0.0019
Scale (y)	1.6e-7±6.9e-9	1.42e-7±3.73e-9	7.57e-8±9.89e-9
n power law	2.37±0.0023	2.91±0.004	2.14±0.0037
Scale power law (C)	0.00058±5.32e-6	9.0e-5±1.45e-6	0.000573±8.32e-6
χ^2	51.91	54.12	10.84
		Deduced parameters	
d_{IT}, Å	709.7	351.4	645.8
d_{TM}, Å	43.6	47.7	38.5
SLD_L, 10^{-6} Å^{-2}	11.85	11.55	11.84
SLD_H, 10^{-6} Å^{-2}	12.73	12.57	12.65

Supplementary Table 6. Lumen composition, expressed as 'relative plastocyanin' content in % (i.e. $\phi_{plastocyanin} + \phi'_{water}$ = 100 %; detailed in *Equation 12*) with different inter-thylakoid space composition scenarios. All feasible lumen compositions depicted in orange, the best composition is in green, second-best is in brown. Disallowed lumen compositions are depicted in red. The best lumen composition is chosen, when $\phi_{plastocyanin}$ values, calculated separately from neutron and X-ray scattering measurements, agree.

**IT space composition**			**Lumen composition**										
Phycobilisome/Water volume fraction inside IT space, % / %	D_2O/H_2O inside IT space, % / %	Labile H-D exchange of phycobilisome, %	100% D_2O + 100% labile H-D exchanged plastocyanin	100% D_2O + 90% labile H-D exchanged plastocyanin	100% D_2O + 50% labile H-D exchanged plastocyanin	100% D_2O + 0% labile H-D exchanged plastocyanin	90%/10% D_2O/H_2O + 90% labile H-D exchanged plastocyanin	90%/10% D_2O/H_2O + 50% labile H-D exchanged plastocyanin	90%/10% D_2O/H_2O + 30% labile H-D exchanged plastocyanin	90%/10% D_2O/H_2O + 0% labile H-D exchanged plastocyanin	80%/20% D_2O/H_2O + 80% labile H-D exchanged plastocyanin	70%/30% D_2O/H_2O + 70% labile H-D exchanged plastocyanin	From X-ray scattering
60/40	100/0	0	59.2	57.1	50.2	43.5	44.7	38.9	36.5	33.5	23.8	-4.8	60.5
70/30	100/0	0	77.1	74.4	65.4	56.7	65.5	57.0	53.5	49.0	49.2	27.0	68.8
80/20	100/0	0	95.1	91.8	80.6	69.9	86.4	**75.1**	70.5	64.6	74.7	58.8	77.0
85/15	100/0	0	104.0	100.4	88.2	76.5	96.8	84.2	79.0	72.4	87.4	74.7	81.2
85/15	50/50	50	113.1	109.2	95.9	83.2	107.3	93.3	87.6	80.2	100.3	90.8	81.2
85/15	80/20	50	82.7	79.9	70.1	60.9	72.1	62.7	58.8	53.9	57.2	36.9	81.2
85/15	90/10	0	108.2	104.4	91.7	79.6	101.6	88.3	**82.9**	76.0	93.3	82.1	81.2
90/10	100/0	0	113.0	109.1	95.8	83.2	107.2	93.2	87.5	80.1	100.1	90.6	85.3

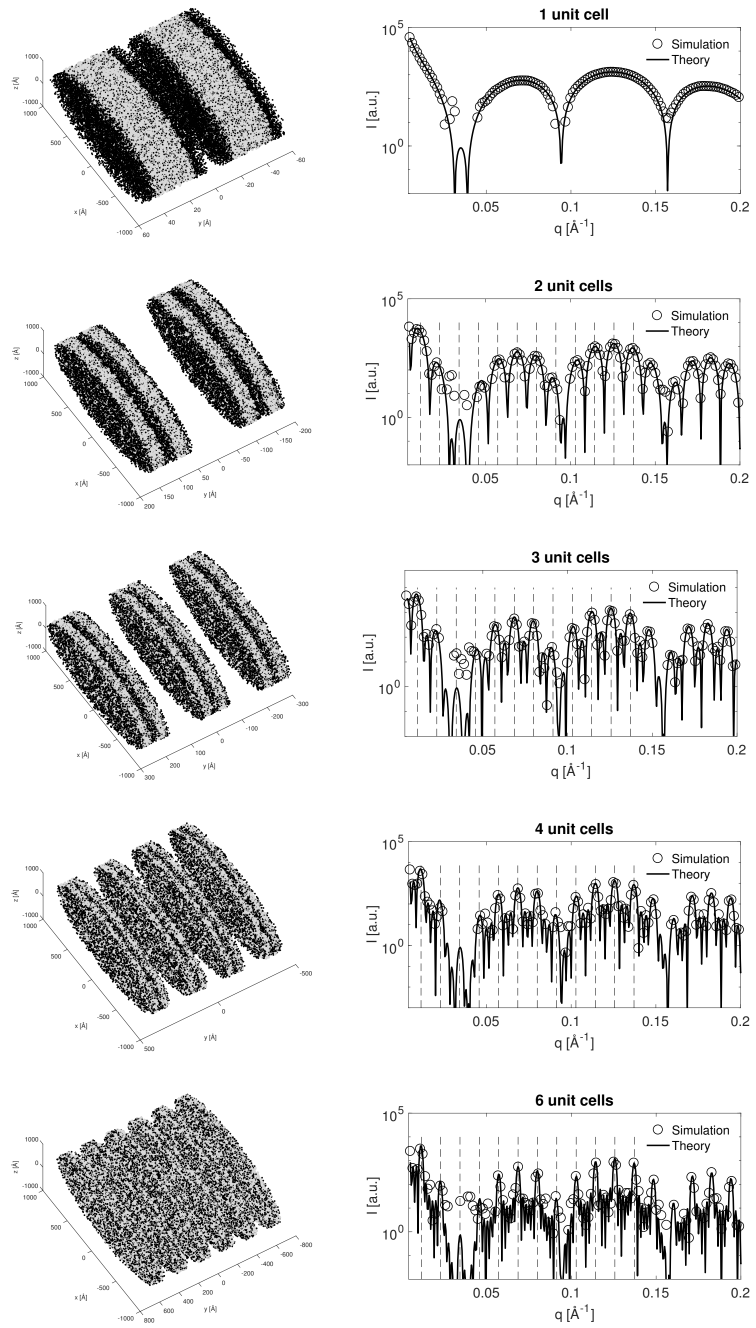

Supplementary Figure 1. In the left column: 3-D point representations of the simulated structures going from 1 to 6 unit cells. Gray points represents the interior bilayer tail region and black points the exterior head group points. Note that the cylinder (y) axis is much smaller than the lateral axes for a unit cell. In the right column: comparing scattering simulations with the theoretical model described in the main text and above. In this example 1-6 unit cells are simulated with parameters: $d_H = 10$ Å, $d_T = 15$ Å, $d_L = 50$ Å, $\rho_H = 0.2$, $\rho_T = -0.1$, $\rho_L = 0$, $RD = 550$ Å and $\eta_{cp} = 0$. N takes values 1-6 as indicated. We have done extensive model calculations to understand the influence of individual model parameters to guide the fitting procedure (not shown). The dashed lines indicate the position of the Bragg peaks of order 1-12 for an infinite perfect stack.

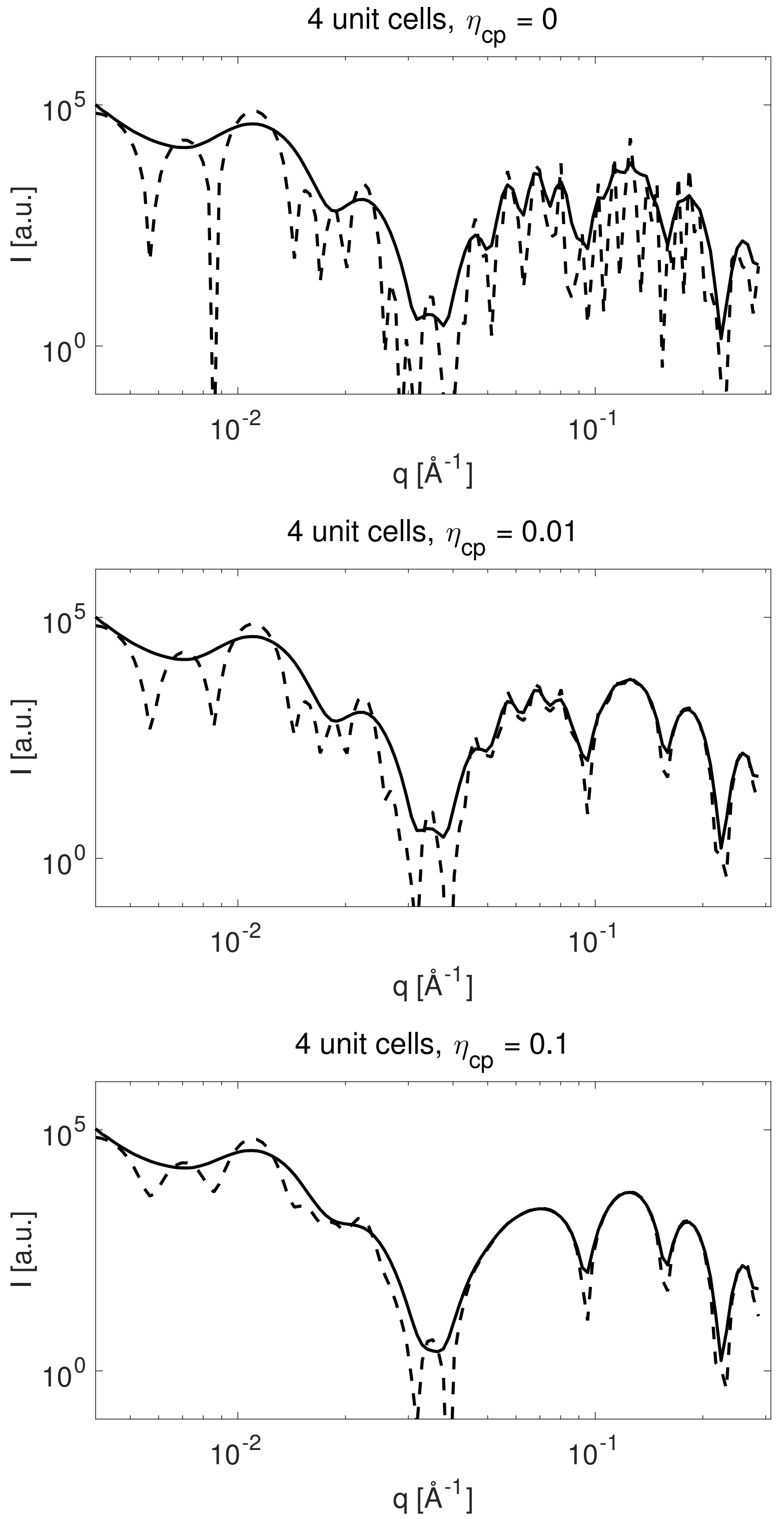

Supplementary Figure 2. Comparing model scattering patterns with (full lines) and without the Bilby instrument resolution smearing (dashed lines). (top) With η_{cp} = 0. Same as Fig. 5(a) in main paper. (middle) With η_{cp} = 0.01. (bottom) With η_{cp} = 0.1. Other parameters as simulations in **Supplementary Figure 1**.

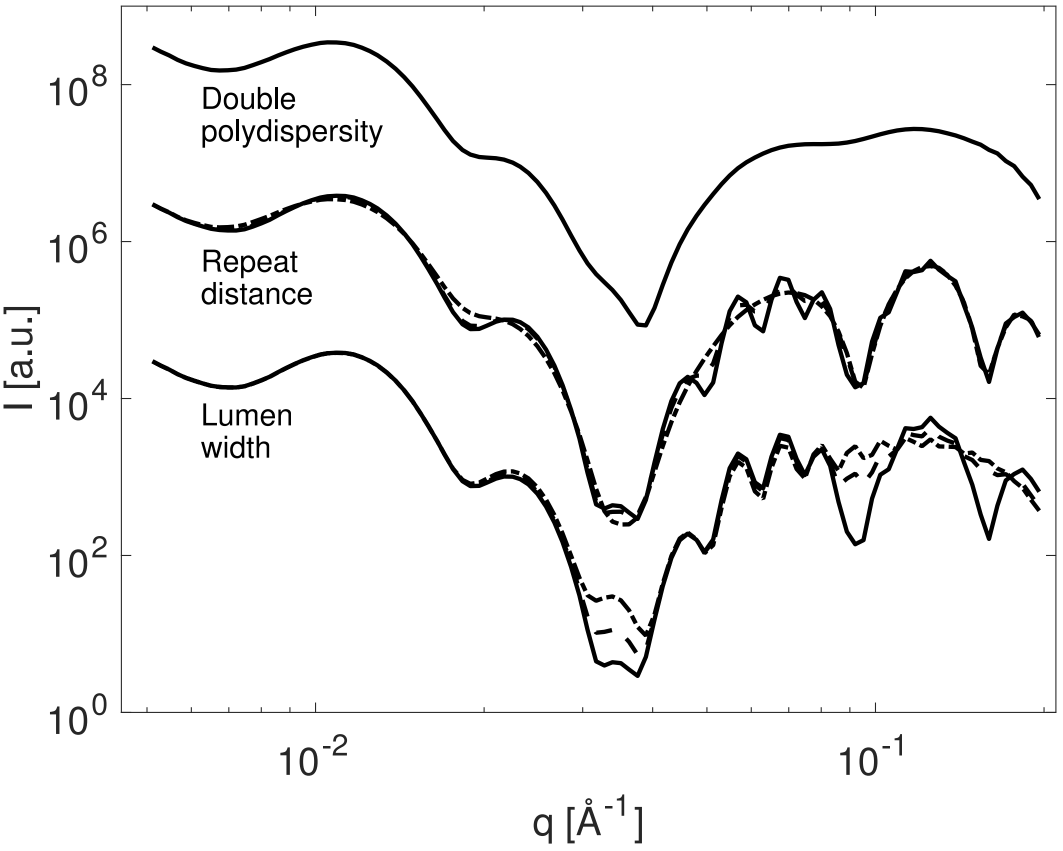

Supplementary Figure 3. Effects of large length scale fluctuations on the scattering patterns. The lower group of curves shows the effect of increasing the lumen width polydispersity σ_L from $0 \rightarrow 10 \rightarrow 20$ Å. The middle curves shows the effect of increasing the repeat distance polydispersity σ_D from $0 \rightarrow 20 \rightarrow 50$ Å. The top curve show the effect of double polydispersity with $\sigma_L = 20$ and $\sigma_D = 50$. All curves have $N = 4$. Other parameters as simulations in **Supplementary Figure 1**.

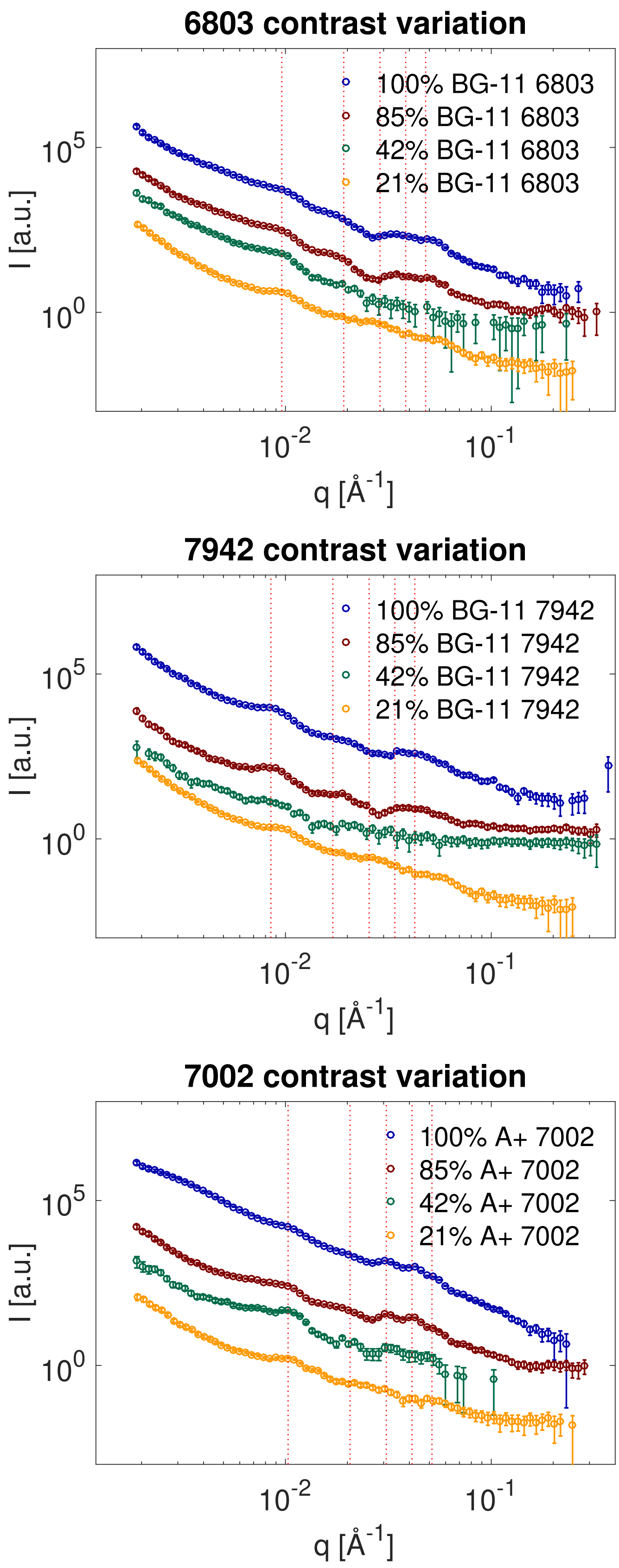

Supplementary Figure 4. Buffer-subtracted contrast variation measures of the investigated cyanobacterial species.

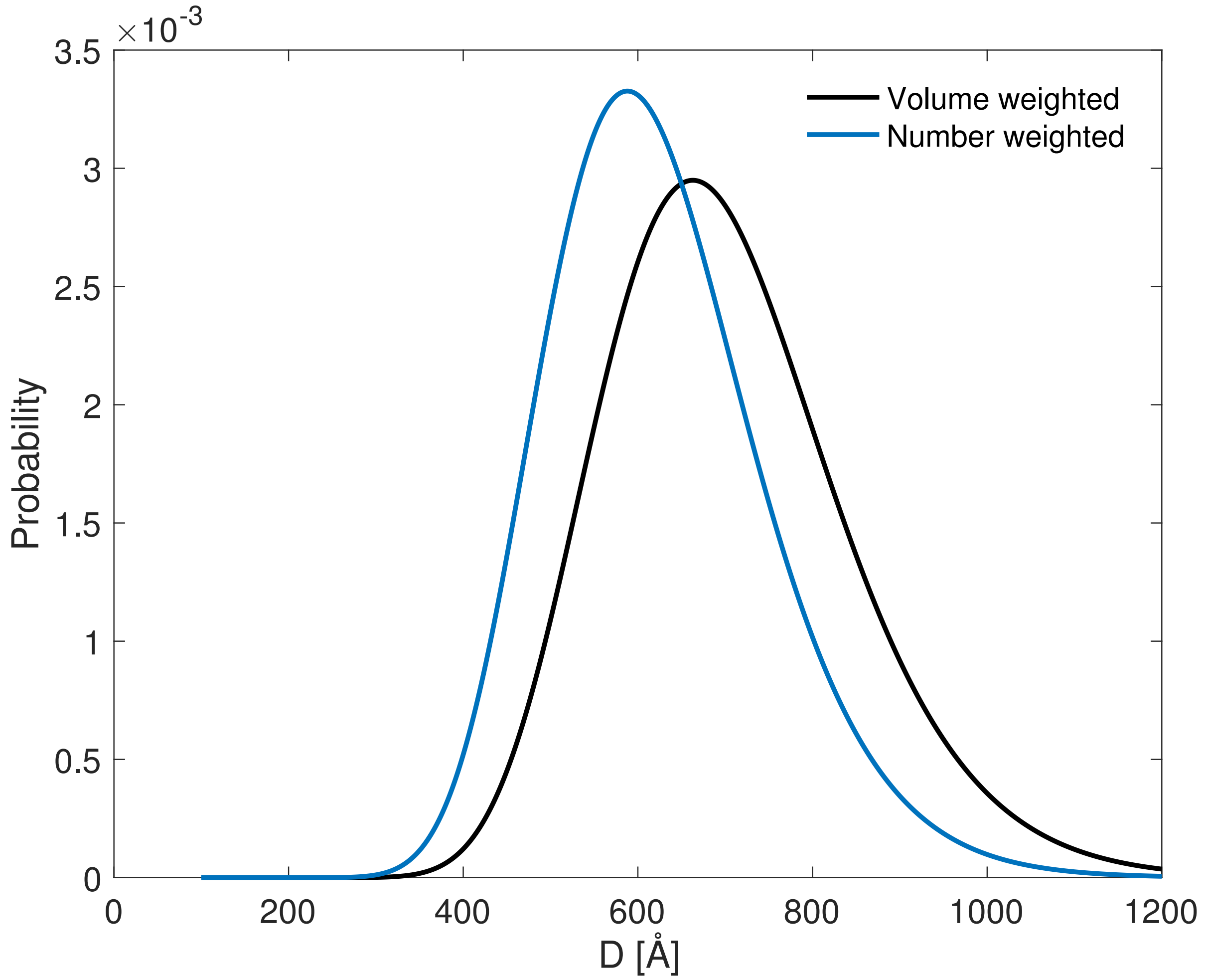

Supplementary Figure 5. Transforming a log-normal probability distribution from volume-averaged to number-averaged. A volume averaged log-normal distribution with mean 690 Å and σ = 0.2 is transformed to a number averaged distribution with mean $\sim$ 590 Å.
